# Large Intrathoracic Desmoid Tumor and Re-Expansion Pulmonary Edema: Case Report and Review of the Literature

**DOI:** 10.3390/medicina58121857

**Published:** 2022-12-16

**Authors:** Efstathia Pistioli, Eleftheria Soulioti, Emmanouil I. Kapetanakis, Thrasyvoulos P. Michos, Periklis I. Tomos, Tatiana Sidiropoulou

**Affiliations:** 1 Second Department of Anesthesiology, National and Kapodistrian University of Athens, Attikon University Hospital, Rimini 1, 12462 Athens, Greece; 2 Department of Thoracic Surgery, National and Kapodistrian University of Athens, Attikon University Hospital, Rimini 1, 12462 Athens, Greece

**Keywords:** re-expansion pulmonary edema, desmoid tumor, lung atelectasis, thoracic surgery, thoracic anesthesia

## Abstract

Re-expansion pulmonary edema is a potentially life-threatening situation following thoracic surgery of a compromised lung. We report the case of a 24-year-old female scheduled for a resection of a large intrathoracic desmoid tumor that presented with re-expansion pulmonary edema at the conclusion of her surgery and discuss the clinical presentation, mechanism and predictors of this entity and review similar cases reported in the literature.

## 1. Introduction

Desmoid tumors are rare clinical entities characterized by aggressive fibromatosis of the connective tissue with mesenchymal characteristics [1]. They can occur in any anatomical location and have a prevalence of 0.03% among all tumors in the general population. Desmoid tumors (DT) tend to compress normal tissue as they proliferate, respecting fascial boundaries [2]. Although DT lack metastatic potential, they have a high propensity for recurrence. Therefore, DT have now been classified as an “intermediate, locally aggressive” tumors in the WHO classification of soft tissue tumors [3].

Re-expansion pulmonary edema (RPE) is an uncommon complication that may occur after treatment and re-inflation of an atelectatic lung [4]. Reported incidence of symptomatic RPE is about 0.5% after thoracentesis [5]. The exact pathophysiological mechanism of RPE has yet to be clarified, but it is believed to be caused by excessive inflammatory response, resulting in increased vessel permeability [6]. We herein describe a case of pulmonary edema complicating the resection of a large desmoid tumor of the thorax.

## 2. Case Report

**Figure 1:**
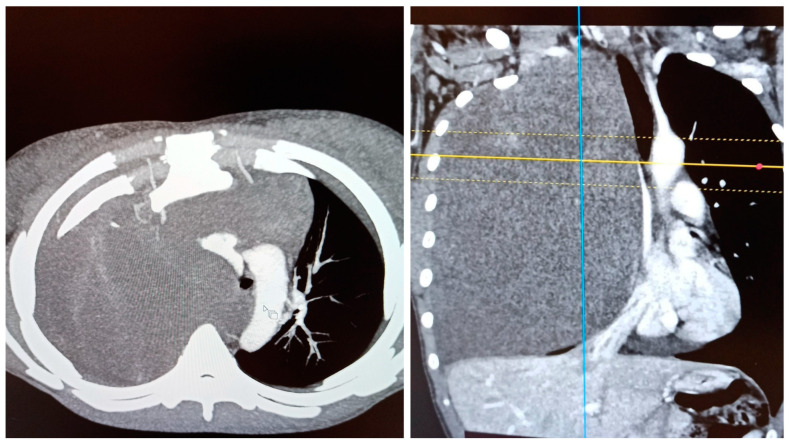
Preoperative CT-scan images of the tumor.

A 24-year-old female (BMI 17.6 kg/m^2^) was scheduled for surgical resection of a desmoid tumor of the right thoracic wall. Diagnosis of the desmoid tumor was confirmed elsewhere via an open biopsy with a mini thoracotomy (Chamberlain procedure) under local anesthesia and sedation. The patient was then referred to our hospital for further treatment. During preoperative evaluation, the patient presented with ongoing dyspnea commencing approximately a year previous and required oxygen therapy with a nasal cannula at 4 L/min, maintaining SpO2 values of 95–96%. Her preoperative arterial blood gas values were pH 7.41, PaCO2 34 mmHg, and PaO2 74 mmHg, with an FiO2 of 36%. Pulmonary function test values were as follows: FVC: 1.85 L (50.3% predicted); FEV1: 1.67 L (51.8% predicted); and FEV1/FVC: 90.1%. The patient’s chest CT-scan revealed a 20 cm × 17 cm × 14 cm mass of the right chest cavity causing atelectasis of the right upper and middle lobe and partial atelectasis of the right lower lobe along with a small pleural effusion (Figure 1).

Her past medical history included a known penicillin allergy, a mild tricuspid valve regurgitation (discovered during her cardiac function evaluation preoperatively), history of recurrent headaches, and iron deficiency anemia. The patient had undergone general anesthesia without any complications at the age of 11 years old for open appendectomy surgery.

Anesthetic management included basic (ECG, SpO2, EtCO2) as well as invasive blood pressure monitoring. Prior to induction of general anesthesia, a thoracic epidural catheter was placed at the T7-T8 interspace for optimal pain management. Induction to general anesthesia was achieved with intravenous midazolam 1 mg, fentanyl 100 mcg, and propofol 150 mg. Intubation was achieved with a 35 Fr left-side double-lumen tube and facilitated with rocuronium 40 mg. Correct placement of the tube was confirmed with flexible bronchoscopy. Maintenance of general anesthesia was achieved with a 1 MAC sevoflurane and bolus doses of ropivacaine 0.375% via the epidural catheter. The patient was ventilated with a tidal volume (Vt) of 5 mL/kg throughout surgery, given the collapsed right lung.

**Figure 2:**
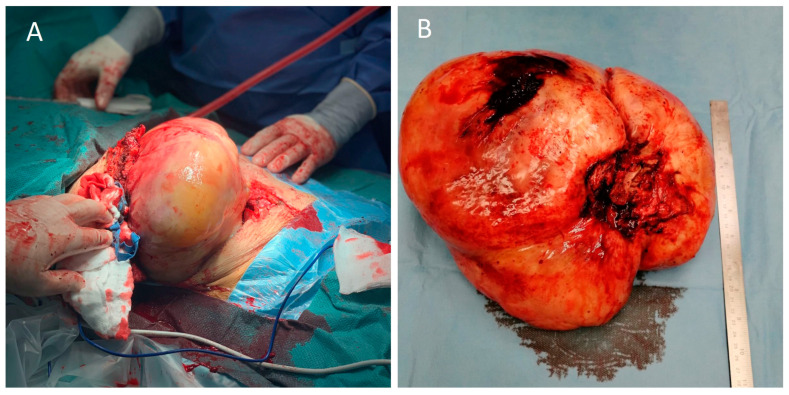
(**A**) Intraoperative image of the resection; (**B**) the resected tumor.

Intraoperatively, access to the intrathoracic mass was obtained through a middle sternotomy and right thoracotomy incisions. Resection included also parts of the 2nd and 3rd rib bodies, where the mass appeared to have originated (Figure 2A,B). Hemodynamic instability during surgery, due to great vessel compression, epidural anesthesia, and hemorrhage was treated with fluids, packed red blood cells (pRBC), and a continuous infusion of intravenous phenylephrine. Surgery lasted 190 min, and one lung ventilation was carried out for 60 min. After removal of the desmoid tumor, slow re-expansion of the previously collapsed right lung was initiated. Our ventilation strategy for lung re-expansion during thoracic surgery includes an alveolar recruitment maneuver with pressure control ventilation and increments of 2 mmHg while peak airway pressure is kept under 30 mmHg. PEEP remains constant and increments of PC are kept for at least 3 breaths. I:E is 1:1 and RR is 6 breaths/min during recruitment. As a consequence of these settings, the alveolar recruitment maneuver lasts approximately 10–15 min. The patient’s gas exchange improved significantly after the tumor removal in comparison to her preoperative status. Post-expansion arterial blood gas values were pH 7.43, PaCO2 33 mmHg, and PaO2 473 mmHg (FiO2 70%).

**Figure 3:**
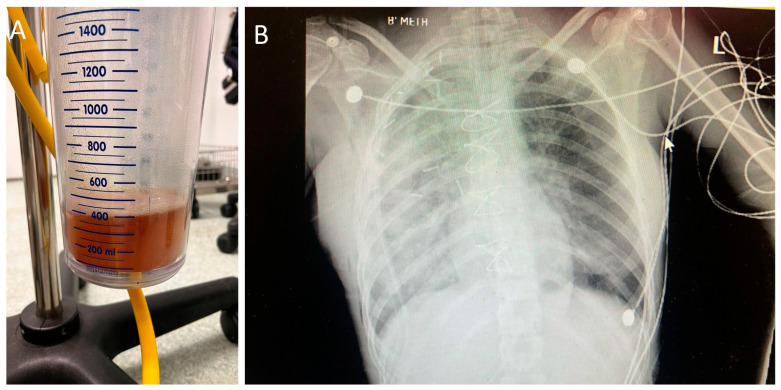
(**A**) Suction contents; (**B**) the patient’s postoperative chest X-ray.

At the conclusion of surgery, the patient had received a total of 2000 mL of intravenous fluids and 2 units of pRBC. Urine output was 600 mL over a 4 h period. The patient was hemodynamically stable, and extubation was attempted. Reversal of neuromuscular blockade was achieved with intravenous sugammadex 2 mg/kg. During awakening, the patient became hypoxic while still intubated and secretions filled the endotracheal tube. Auscultation revealed diffuse moist rales of the right lung. Suctioning of the right lung was copious, and 400 mL of serous fluid was collected (Figure 3A). Re-expansion pulmonary edema was suspected, and the patient was sedated again. The two-lumen tube was exchanged with a single lumen tube, and the patient was transferred to the intensive care unit (ICU) for postoperative mechanical ventilation. Chest X-ray at the ICU revealed diffuse alveolar infiltrates of the right lung (Figure 3B). The patient remained sedated for 24 h post-operatively where she was treated with continuous positive pressure ventilation and administration of corticosteroids (cortisol 50 mg, 4/day, reduced to twice/day on the 4th postoperative day) and diuretics (furosemide 10 mg once/day). The patient’s status improved significantly, and she was successfully extubated after 30 h post-surgery and treated with non-invasive ventilation for 2 more days. She remained in the ward for 5 more days and was discharged from the hospital with no further complications. Postoperative histological examination of the mass confirmed the diagnosis of a desmoid tumor. Surgical excision margins were clear of tumor cells.

## 3. Discussion

Desmoid tumors are mostly located abdominally, while the chest wall represents only 8–10% of their location [3,7]. Patients usually present with a peak age of 30–40 years [3,7]. The resection of a large desmoid tumor of the chest cavity, with consequent occurrence of RPE, represents an exceedingly rare event. The anesthetic management is challenging, as the onset is rapid and unexpected, with a dramatic clinical appearance, while the pathophysiology can be unclear. There is paucity in the literature regarding similar cases of desmoid tumors complicated with RPE. A retrospective study by Kabiri et al. that included eight patients (mean age 32 years) over a period of 20 years who underwent chest wall desmoid tumor resection reported no incidence of RPE [8]. Similarly, Iqbal et al. reported no RPE after surgical removal of an intrathoracic desmoid tumor [9].

Only a few cases of tumor resection complicated with RPE have been reported. Yu et al. reported the case of a 55-year-old patient with bronchial and cerebellar lesions [10]. Surgical excision of the cerebellar lesion was carried out, which was complicated with RPE, presenting with foam sputum during suctioning and hypoxia before extubation. The patient was transferred to the ICU and was discharged at the eighth post-operative day. Havránková et al. reported the case of a 33-yea-old male scheduled for a mediastinal tumor resection [11]. After tumor removal, complications including circulatory shock and undefined hemorrhage lead to the patients’ intraoperative death, despite resuscitation efforts. The authors suspected that RPE was the reason. Yanagidate et al. reported the case of a 74-year-old male patient scheduled for left thoracoscopic mediastinal tumor resection and right lower lobectomy for squamous cell carcinoma [12]. At the end of the first surgical procedure, after changing from right to left lateral position, RPE of the left lung occurred. Matsumiya et al. describe the case of a 17-year-old male patient that presented with RPE in the operating room after the resection of a mediastinal tumor and who required stabilization and mechanical ventilation, extubated the day after in the ICU [13]. Sagara et al. reported RPE immediately after the resection of a large mesothelioma in a 70-year-old female patient, and Otomo et al. in a 64-year-old female patient following the removal of a giant thoracic tumor with associated mediastinal shift [14,15]. A 50-year-old female patient that underwent an intraabdominal giant tumor resection manifested RPE at the end of the surgery, according to Mihara et al. [16]. Similarly, Hari et al. described the case of a 19-year-old female patient affected by anorexia nervosa and a large ovarian tumor [17]. Before extubation, she also presented with RPE, was transferred to the ICU, and extubated 12 h post-surgery.

From these cases reported in the bibliography, the onset of RPE occurs mainly at the end of the surgery, as was observed also in our case. Despite slow, progressive pulmonary insufflation in our patient, RPE of the atelectatic lung occurred. A possible explanation may rest on the premise that the tumor was compressing the lung for a prolonged time, approximately 2 years. During this time the patient manifested progressive dyspnea and required oxygen therapy to maintain adequate respiratory function. In fact, predisposing factors for RPE include the patients’ young age, the duration and extent of lung involvement, the rapid re-expansion of the lungs, and surfactant dysfunction [9,10,11].

Although the underlying mechanism of RPE is still unclear, increased permeability of the vascular endothelium and altered architecture of the lung microvascular bed seem to have a leading role in RPE occurrence [9,10,11,18]. Chemotactic factors such as interleukin-8 (IL-8), monocyte chemotactic protein-1 (MCP-1), and pulmonary surfactant may also be involved [19]. It is hypothesized that the pulmonary microvascular endothelium becomes thickened and less flexible during chronic lung collapse. When the lung is subsequently re-expanded, shear stress and ischemia-reperfusion injury result in the mechanical destruction of pulmonary microvessels [19,20]. Such mechanical and ischemic injuries are thought to be caused by the secretion of IL-8 and MCP-1, leading to increased permeability of the pulmonary vascular bed.

Other authors propose that, together with the abovementioned factors, heart–lung interactions may also play a role in the pathogenesis of RPE [21]. As suggested, increased pleural pressure (due to the presence of the tumor in our case) decreases the transmural pressure of the left ventricle (LV), therefore decreasing the ejection pressure, which will in turn lead to decreased pressures upstream. When the obstruction is removed, following tumor resection, the subsequent decrease in all pressures involved results in increased LV afterload, with subsequent LV failure and pulmonary edema [21].

To conclude, RPE during surgery is a serious and life-threatening condition. The anesthetic management is of major importance, in order not only to keep the patient alive but to facilitate stabilization and extubation in the hours following surgery. Long duration of lung injury and microvascular pulmonary dysfunction predispose one to RPE. Prevention is always better than treatment, so gentle and slow re-inflation of the deflated lung is necessary. Further research in the fields of pathophysiology of RPE is required, so that a clarified mechanism can lead to safer peri-operative recommendations.
